# Detecting different pesticide residues on Hami melon surface using hyperspectral imaging combined with 1D-CNN and information fusion

**DOI:** 10.3389/fpls.2023.1105601

**Published:** 2023-05-08

**Authors:** Yating Hu, Benxue Ma, Huting Wang, Yuanjia Zhang, Yujie Li, Guowei Yu

**Affiliations:** ^1^ College of Mechanical and Electrical Engineering, Shihezi University, Shihezi, China; ^2^ Key Laboratory of Northwest Agricultural Equipment, Ministry of Agriculture and Rural Affairs, Shihezi, China; ^3^ Engineering Research Center for Production Mechanization of Oasis Characteristic Cash Crop, Ministry of Education, Shihezi, China

**Keywords:** hyperspectral imaging, pesticide residues, convolutional neural network, information fusion, non-destructive detection

## Abstract

Efficient, rapid, and non-destructive detection of pesticide residues in fruits and vegetables is essential for food safety. The visible/near infrared (VNIR) and short-wave infrared (SWIR) hyperspectral imaging (HSI) systems were used to detect different types of pesticide residues on the surface of Hami melon. Taking four pesticides commonly used in Hami melon as the object, the effectiveness of single-band spectral range and information fusion in the classification of different pesticides was compared. The results showed that the classification effect of pesticide residues was better by using the spectral range after information fusion. Then, a custom multi-branch one-dimensional convolutional neural network (1D-CNN) model with the attention mechanism was proposed and compared with the traditional machine learning classification model K-nearest neighbor (KNN) algorithm and random forest (RF). The traditional machine learning classification model accuracy of both models was over 80.00%. However, the classification results using the proposed 1D-CNN were more satisfactory. After the full spectrum data was fused, it was input into the 1D-CNN model, and its accuracy, precision, recall, and F1-score value were 94.00%, 94.06%, 94.00%, and 0.9396, respectively. This study showed that both VNIR and SWIR hyperspectral imaging combined with a classification model could non-destructively detect different pesticide residues on the surface of Hami melon. The classification result using the SWIR spectrum was better than that using the VNIR spectrum, and the classification result using the information fusion spectrum was better than that using SWIR. This study can provide a valuable reference for the non-destructive detection of pesticide residues on the surface of other large, thick-skinned fruits.

## 1 Introduction

Fruits and vegetables are a significant part of people’s daily diet, providing basic vitamins, edible fiber and minerals, and a large amount of protein, carbohydrates, and other nutrients. Xinjiang is a famous Hami melon producing area in China, which has the natural climatic conditions required for the growth of Hami melon. However, Hami melon is susceptible to various pests and diseases during the growing season, such as powdery mildew, bacterial leaf spot disease, anthrax, and aphids, resulting in reduced yields. Farmers generally use fungal fungicides and insecticides for disease control. The rational use of pesticides can effectively prevent and control pests and diseases, but improper use can cause pesticide residues on the surface of fruits and vegetables. Some farmers lack common sense in the use of pesticides and unilaterally pursue high yields, making the irregular use of pesticides, abuse, and other problems increasingly severe. Moreover, because Hami melon is large and has a lot of surface reticulation, it is more likely to cause pesticide residues on the surface. With the emphasis on food safety, it is, therefore, necessary to accurately detect pesticide residues on the surface of Hami melon. The traditional chemical detection methods for pesticide residues mainly include Ultrahigh-Performance Liquid Chromatography–Hybrid, immunoassay, and Gas Chromatography-Mass Spectrometry (Garcia-Febrero et al., 2014; Liu et al., 2021; Jia et al., 2022). Chemical detection methods for pesticide residues have high accuracy and sensitivity. Still, most of them are destructive and have the disadvantages of relying on sample pretreatment, slow detection speed, and high detection prices.

Hyperspectral imaging (HSI) is a rapid, non-destructive detection technology. It can obtain the spatial distribution spectrum information of each pixel of the tested sample; that is, it can obtain three-dimensional mapping data information, considered an effective technology to solve the defects mentioned above of traditional chemical detection methods. Currently, HSI technology has been widely used in the quality and safety detection of agricultural products (Li et al., 2018; Yao et al., 2022). Some researchers measured the content of soluble solids in fruits by hyperspectral imaging technology (Li et al., 2016a; Gao et al., 2022; Xu et al., 2022b). Furthermore, some researchers detected aflatoxin in corn, almond, and other crops by HSI technology (Chu et al., 2017; Kimuli et al., 2018; Mishra et al., 2022). Besides, HSI technology was also applied to the non-destructive detection of crop pests and diseases (López-Maestresalas et al., 2016; Lee et al., 2016). In recent years, relevant research has shown that HSI technology has also been widely used to detect pesticide residues on the surface of fruits and vegetables. Some researchers have used near-infrared hyperspectral imaging (NIR-HSI) systems for the qualitative and quantitative detection and analysis of pesticide residues on the surface of lettuce leaves, achieving non-destructive and accurate identification of multiple pesticide residues in lettuce leaves (Sun et al., 2018; Cong et al., 2021). Gui et al. (2018) sprayed water and three pesticides (imidacloprid, abamectin, and propineb) on the surface of broccoli and used HSI technology and machine learning to complete the classification of four categories of pesticide residues. The extreme learning machine (ELM) model using the continuous projection algorithm got the best recognition results, with the accuracy of 98.33% and 96.67% in the training and test sets, respectively. Meanwhile, some researchers used the HSI technique to separately detect different pesticide residues and residue levels on spinach leaves (Ji et al., 2018; Ren et al., 2018). Other researchers also used NIR-HSI to detect pesticide residues in mulberry leaves and to visualize their distribution (Jiang et al., 2017). Li et al. (2016b) used HSI technology to detect pesticide residues with different concentrations on the orange surface. The above literature, showed that the HSI technology has been widely used for the non-destructive detection of pesticide residues on the surface of fruits and vegetables. However, there is no research on pesticide residues in large, thick-skinned melons. Meanwhile, most of the related studies have been performed using a single band range of spectral regions for detection. However, the spectral responses of different band ranges are not the same. Since the two spectral regions may have complementary information, it is proposed to explore the fusion of the spectral information of the two bands and to develop a qualitative discriminative depth model for pesticide residues on the surface of Hami melon.

HSI can provide a large number of features, including spectral features and spatial features. As high-dimensional data, HSI has a large amount of data information, but it is still a difficult task to mine information effectively. Deep learning is currently a prevalent data processing method with a wide range of applications in the field of data processing. Deep learning can learn features quickly and profoundly and efficiently process large amounts of data (Signoroni et al., 2019). Its most significant advantage is that it is deep enough, and the network capacity is large enough. Several researchers have used Convolutional Neural Networks (CNN) to process HSI for detecting agricultural products, crop quality and safety. Al-Sarayreh et al. (2020) studied the potential of HSI and deep learning (3D-CNN) methods in meat classification, and for NIR-HSI and VIS-HSI, the accuracy of classification was 96.90% and 97.10%, respectively. Good results were obtained by Yan et al. (2021) using a visible/near infrared (VNIR) HSI system and CNN to identify aphid infection in cotton leaves. Polder et al. (2019) combined HSI and deep learning for the non-destructive detection of potato seed viruses with accuracy and recall exceeding 78.00% and 88.00%, respectively. In addition, deep learning is also commonly used to extract effective features of spectral data for modeling classification or regression. Jiang et al. (2022b) used hyperspectral as well as discrete wavelet transform and deep learning modeling methods for detecting and identifying veterinary drug residues in beef, using deep learning for modeling to shorten the prediction time and greatly improve the accuracy of classification and identification. Meanwhile, some researchers have also started combining CNN with HSI to detect pesticide residues on the surface of fruits and vegetables. Jiang et al. (2022a) used hyperspectral technology for the detection of pesticide residues on cabbage, classifying no residues as well as four pesticides (chlorpyrifos, cypermethrin, methomyl, and dimethoate), and found that the fusion of hyperspectral with the discrete wavelet transform and CNN could significantly improve the classification recognition accuracy, with a recognition accuracy of 91.20%. Ye et al. (2022) used HSI combined with CNN to detect pesticide residue levels in grapes containing no residues and three different gradients of pesticide residues, and obtained good results. There was also a related researcher who used a short-wave infrared (SWIR) hyperspectral imaging system to detect mixed pesticides on the surface of leek leaves and classified them using 1D-CNN with a test set accuracy of 97.90% (He et al., 2021). As seen in the above article, the deep learning performed well in HSI and spectral data. This indicated that deep learning can indeed eliminate the need for upfront feature analysis and extraction, and it did improve as the amount of information increased, the depth of the network deepened, and appropriate optimization mechanisms were added.

As “pollution-free” and “green” have become important criteria for consumers to choose fruit and vegetable products, it is urgent to solve the problem of rapid non-destructive detection of pesticide residues on the surface of Hami melon. While most of the traditional chemical detection methods are destructive, hyperspectral imaging has great potential for the non-destructive detection of pesticide residues on the surface of fruits and vegetables, but few studies have been conducted for large melons. In addition, convolutional neural network models reduce the need for manual feature engineering and provide a new method for constructing qualitative pesticide residue discrimination models with high accuracy and generalization capability. Information fusion techniques provide a technique that can effectively enhance the effectiveness of models (Hong et al., 2021a, 2021b). Therefore, the study took Xinjiang specialty fruit Hami melon as the carrier, selected commonly used pesticides as the research object, and explored the feasibility of using hyperspectral imaging technology and convolutional neural network model for non-destructive detection of pesticide residues on the surface of Hami melon.

This study aims to use HSI combined with deep learning to identify different species of pesticide residues on the surface of Hami melon. The specific objectives are: (1) to investigate the spectral differences of different species of pesticide residues in different band ranges; (2) to compare the performance of hyperspectral imaging in single and fused band ranges for pesticide residue species identification; (3) to develop a multi-branch one-dimensional convolutional neural network (1D-CNN) model with attention mechanism for discriminating different species of pesticide residues on the surface of Hami melon; (4) to compare the classification performance of a multi-branch 1D-CNN model with attention mechanism model with the traditional 1D-CNN classification model; (5) to compare the classification performance of a multi-branch 1D-CNN model with attention mechanism model with traditional chemometric classification models in terms of their classification performance.

## 2 Materials and methods

### 2.1 Sample preparation

The same batch of Hami melon (Xizhoumi No. 25) was purchased from Shihezi Agricultural Products Trading Center in Xinjiang, China, and transported to Shihezi University for sample preparation. 200 Hami melons with oval shapes weighing about 3-4 kg were randomly selected. Four standard pesticides were purchased from the local agricultural raw materials market in Shihezi, Xinjiang, China. They were Acetamiprid (Active ingredient content 70%, water dispersible granule, Shandong Baixin Biotechnology Co., Ltd., China.), Malathion (Active ingredient 70%, emulsifiable oil, Ningbo Sanjiang Yinong Chemical Co., LTD., China), Difenoconazole (Active ingredient 20%, microemulsion, Chengdu Kelilong Biochemical Co., LTD., China) and Beta-cypermethrin (Active ingredient 4.5%, emulsion, Jiangsu Yixing Xingnong Chemical Products Co., LTD., China), respectively.

All Hami melon samples were wiped and numbered before data collection. Then put them in a well-ventilated laboratory (indoor temperature 22°C, relative humidity 40%) and let them stand for 24 hours to reduce the influence of environmental factors on the model accuracy. Two hundred samples were randomly divided into five equal groups (40 samples in each group). The above four pesticides were mixed with distilled water to prepare pesticide solutions with a ratio of 1:1000, which were evenly sprayed on the surface of Hami melon, and recorded as group 1, group 2, group 3, and group 4. In addition, the remaining 40 Hami melon samples were used as the control group, and distilled water was evenly sprayed on the surface of each sample, recorded as group 0. Finally, all prepared samples were stored indoors for 12 hours.

### 2.2 Hyperspectral imaging acquisition

#### 2.2.1 Hyperspectral imaging system

**Figure 1:**
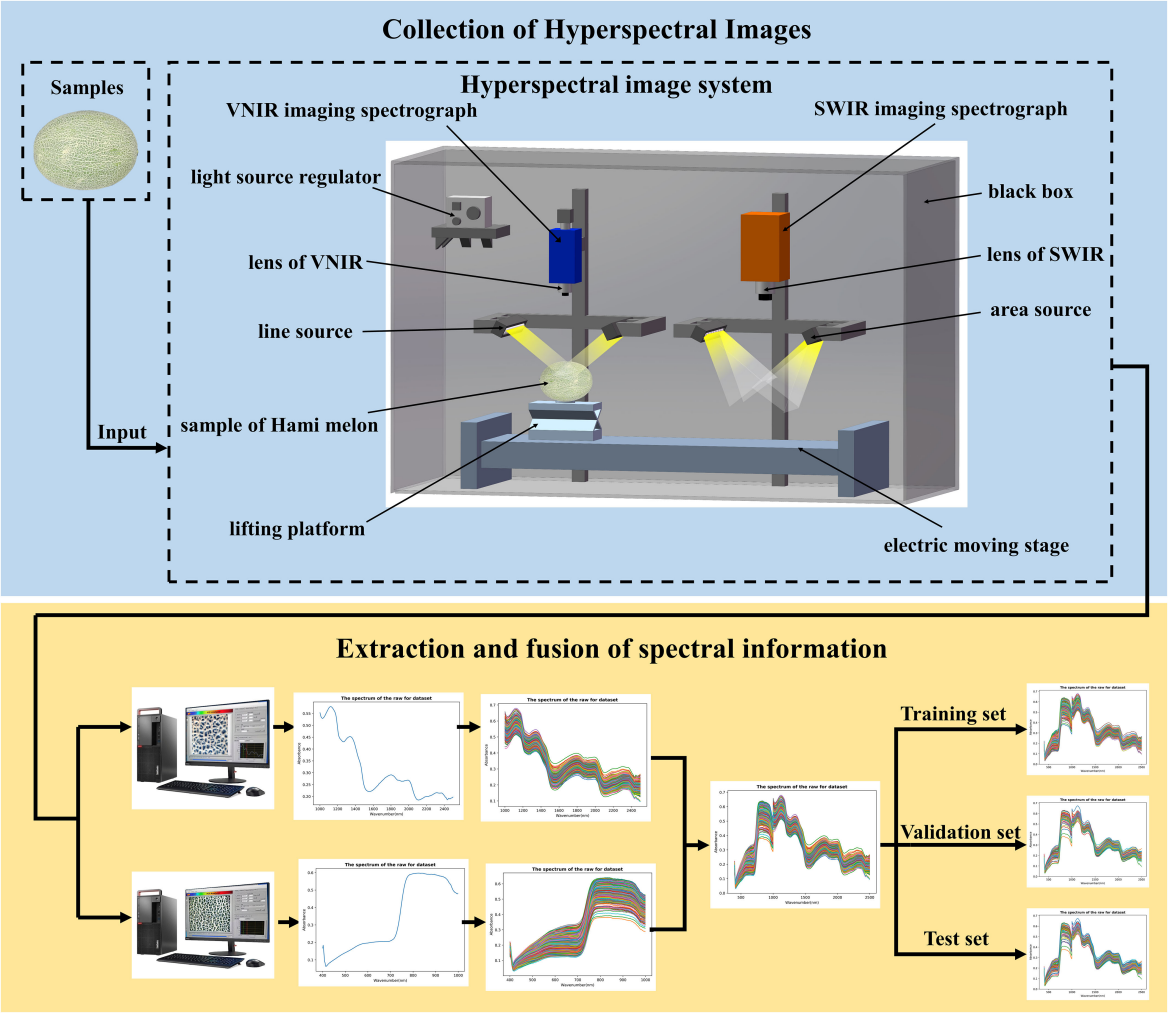
Collection of hyperspectral images and division of data sets.

In this study, the hyperspectral imaging system consisted of VNIR and SWIR hyperspectral imaging systems. VNIR hyperspectral imaging instruments included an imaging spectrometer (ImSpector V10E2/3^’’, Specim, Oulu, Finland), a high-resolution camera (GEV-B1621M, Photon Etc., Montreal, Canada), and two halogen line light sources. SWIR hyperspectral imaging instrument consisted of an imaging spectrometer (ImSpector N25E2/3^’’, Specim, Oulu, Finland), a high-resolution camera (Zephir-2.5–320, Photon Etc., Montreal, Canada), two 150W halogen surface light sources. Both line and surface sources were angled at about 45° to illuminate the field of view below the spectrometer. Two devices shared an electrically positioned sample stage operated by a stepper motor. When collecting hyperspectral images, two hyperspectral images with different wave bands can be scanned with the same attitude. All the above instruments were placed in a box with a black inner surface to form the hyperspectral imaging system. In addition, a computer equipped with data acquisition software (Spectral Image System, Isuzu Optics Corp., Taiwan, China) was provided. The hyperspectral imaging system is shown in **Figure 1**. Hyperspectral images of Hami melon samples containing pesticide residues were obtained in diffuse reflection mode. The measurement was carried out in a dark room to avoid light interference. In addition, the hyperspectral imaging system is shown in **Figure 1**.

#### 2.2.2 Imaging acquisition and calibration

Proper adjustment of the acquisition parameters is vital to obtain clear and distortionless hyperspectral images. In this study, the spectral variability was avoided by setting appropriate parameters (Hong et al., 2019). The distance between the upper surface of Hami melon samples and the CCD camera lens was set to 19.7 cm. In this study, the correlation coefficient of VNIR-HSI was set as follows. The camera’s exposure time and the speed of the electric moving stage were adjusted to 6.5 ms and 0.803 mm/s, respectively. The correlation coefficient of SWIR-HSI was set as follows. The camera’s exposure time and the speed of the electric moving stage were adjusted to 4.1 ms and 53.4 mm/s, respectively.

The instrument needs to be preheated for 30 minutes before use. As VNIR and SWIR were assembled in one system, with the movement of the electric mobile station, the acquisition system could simultaneously acquire the images and spectral information of the Hami melon samples in two wave bands and repeat the operation until all the sample information was obtained. In fact, the directly obtained hyperspectral images of Hami melon cannot be immediately used in experiments. They need to be black, and white corrected first. To reduce the influence caused by uneven light source intensity distribution and CCD camera dark current noise, the raw intensity image (R_r_) was calibrated into reflectance images using white and dark references. The white reference image (R_w_) was acquired using a white Teflon bar with nearly 99% reflectance, and the dark reference image (R_d_) was obtained by turning off the light source and closing the lens cover. The final corrected image (R_c_) was calculated according to the following Equation (1):


(1) $$R_{c}=\frac{R_{r}-R_{d}}{R_{w}-R_{d}}$$

### 2.3 Data analysis

#### 2.3.1 Spectral data extraction

The image resolution of the VNIR-HSI camera is 1632×1232 pixels, and the spectral resolution is 2.03 nm. The spectral range is 374.2847-1033.048 nm, which can collect hyperspectral images of 308 bands. The image resolution of the SWIR-HSI camera is 320×256 pixels, and the spectral resolution is 6.20 nm. The spectral range is 982.38-2618.37 nm, which can collect hyperspectral images of 288 bands. After edge band screening, the obtained bands ranges were 400-1000 nm and 1000-2500 nm, respectively, and the spectral data of 279 and 233 bands were retained. For each Hami melon, one hyperspectral image was collected every 90° rotation along the equatorial direction. So, four hyperspectral images can be collected from one Hami melon sample, and 800 hyperspectral images can be collected from 200 Hami melon samples. This hyperspectral imaging system could collect data from two bands simultaneously, and then 1600 hyperspectral images could be collected. After the hyperspectral image was collected, the hyperspectral image was imported into ENVI 5.3 (Exelis Visual Information Solutions, USA), and pixel blocks of 50×50 at the equatorial position of Hami melon were randomly selected as regions of interest. A total of 1600 average spectra information were collected in the two bands, which were stored to establish the classification model. That is, the spectral data was extracted from the collected hyperspectral images, and the model was trained and verified by using the data differences between spectral information, so as to quickly and effectively identify different kinds of pesticide residues. The sample data were randomly divided into training, validation, and test sets with a ratio of 5:1:2. In addition, the division of the data set is shown in **Figure 1**.

#### 2.3.2 Information fusion

Information fusion, which can be called data fusion or multi-sensor information fusion, is a multi-level and multi-faceted process. Compared with the single detection technology, the information fusion technology has the advantages of more information and better fault tolerance. Information fusion can realize the fusion process of multi-source information at multiple levels. According to the level of data abstraction in the fusion system, the fusion can be divided into data layer fusion, feature layer fusion, and decision layer fusion.

Data layer fusion refers to directly associating the original data of each sensor and sending it to the fusion center to complete the comprehensive evaluation of the measured object. Feature layer fusion means that the original data is processed by feature extraction, correlation, and normalization and then sent to the fusion center for analysis and synthesis, to complete the tested object’s comprehensive evaluation. Decision layer fusion means that the signals from each sensor are processed locally before fusion. Each sensor’s corresponding processing unit first performs tasks such as feature extraction and decision-making separately and independently. Then correlated and sent them to the fusion center for processing.

When the recognition results are fused at the decision layer, it is difficult to measure the weights of the two sensors. And it needs a target knowledge base and massive data preprocessing, which leads to information loss. The feature layer fusion method may be more suitable for fusing two different types of sensor data. For data layer fusion, it needs the requirement of consubstantial sensors, and the data layer fusion method has the minimum information loss and high fusion accuracy (Huang et al., 2014). At the same time, the convolution neural network is used to process and classify the data, which solves the problems of information redundancy and a large amount of calculation. In this study, the data layer fusion method was used for information fusion. In addition, the spectral curve of data layer fusion is shown in **Figure 1**.

#### 2.3.3 Spectral data preprocessing

The spectral data information of the two bands expresses the physical meaning of the spectral reflection value numerically. However, considering their different numerical ranges, they need to be normalized before modeling and analysis. As a data preprocessing method, maximum-minimum normalization can limit the data to be processed in a certain range. It can ensure convergence speed in the process of the program running and provide convenience for subsequent data processing.

The spectrum needs to be preprocessed after the maximum-minimum normalization. Raw spectral data usually carry some information and noise unrelated to the composition of the sample. Through spectral preprocessing, noise and background interference can be removed appropriately, thus significantly improving the performance of the model. Before modeling, it is necessary to preprocess the original spectrum. All raw spectral data were preprocessed by Savitzky-Golay (S-G) (Savitzky and Golay, 1964). The preprocessing treatment was implemented using MATLAB R2019b (The MathWorks, Natick, MA, USA).

#### 2.3.4 Optimal band selection

Raw spectra in the entire band range and preprocessed spectral data contain large amounts of redundant information. In many cases, better classification results can be obtained using the optimal band rather than the whole band range. When building a traditional machine learning classification model, if more spectral information in the model is not related to the pesticide residues on the surface of Hami melon, it will affect the model’s accuracy and calculation speed. The selection of spectral characteristic bands has a good influence on subsequent modeling and classification. In this study, the genetic algorithm (GA) (Goldberg, 1989) was used to extract characteristic bands to simplify the model.

The GA simulates the natural selection mechanism of Darwinian biological evolution through the evolutionary process of genetic mechanisms. Using this approach, each spectral data band represents a gene on a chromosome; an encoding of ‘1’ means that the band is selected, whereas a ‘0’ means it is not selected. Each chromosome corresponds to a feature selection scheme. In this study, the initial population size was set to 50, and individuals were randomly selected by computer. In the population, the fitness value of each chromosome was calculated. Chromosomes with high fitness values were retained for replication, crossover, and mutation. Mutation probability, crossover probability, and iteration number were set to 0.01, 0.5 and 100, respectively.

### 2.4 Classification algorithms

#### 2.4.1 KNN

The K-nearest neighbor (KNN) algorithm is a classification algorithm in supervised learning that performs classification by measuring the distance between different feature values (Abeywickrama et al., 2016). It works by partitioning the feature vector space using training data and using the partitioning results as the final algorithmic model. K objects are randomly selected and assigned to the nearest (most similar) group based on their distance from the initial mean of each group, and then the new mean of each group is recalculated. This process is repeated until all objects have found their nearest group in the K-group distribution. K was set to 3 in this study.

#### 2.4.2 RF

Random forest (RF) is a classification model that uses multiple trees to train and predict samples (Witten et al., 2017). It is suitable for high-dimensional and large numbers of samples when preparing data. Hence, its advantage lies in its excellent performance in handling high-dimensional and large numbers of data. In addition, the random forest does not require any special tuning during training. Still, it only requiers an appropriate increase or decrease of the tree, which is more effective in improving the accuracy. In this study, the n_estimator was set to 100.

#### 2.4.3 Multi branch 1D-CNN model with attention mechanism

**Figure 2:**
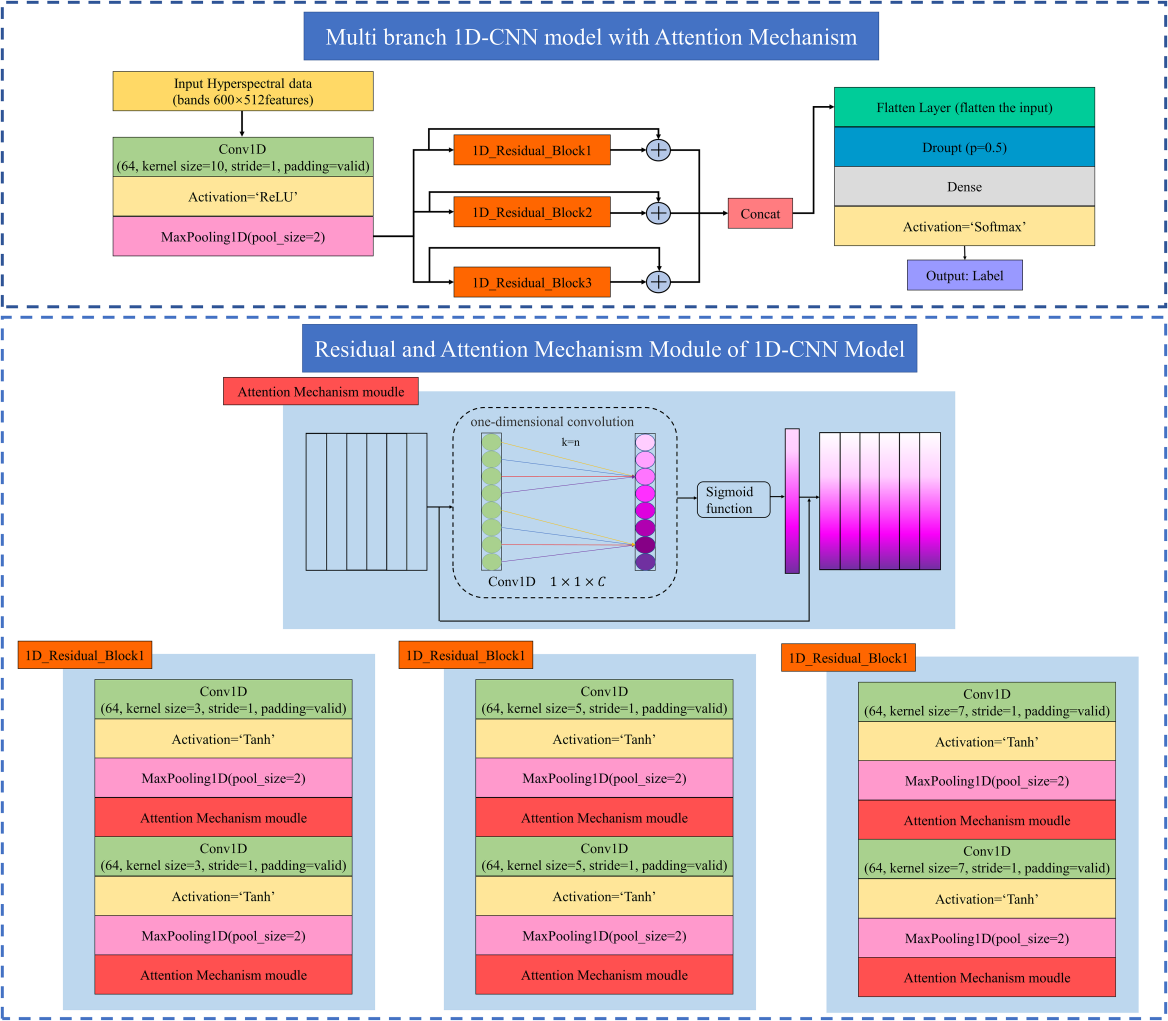
Multi branch 1D-CNN model with Attention Mechanism.

Our study proposed a multi-branch 1D-CNN architecture with an attention mechanism. The specific structure is shown in **Figure 2**. The model was divided into three main parts.

First, the spectral data after normalization and smoothing denoising preprocessing were input to the model, involving 1D spectral data, so Conv1D was used. The spectral data was passed through a convolutional layer, which was then activated by a rectified linear unit (ReLU) function to complete the non-linear transformation of the features. Finally, a maximum pooling layer was added to reduce the over-sensitivity of the convolutional layer. To expand low-bit data to high-dimensional data and facilitate feature extraction later, set the parameters as follows. The input channel of the convolutional layer was 64, the kernel size was 10, and the step size was 1. The maximum pooling layer kernel size was 2.

The data was then fed into the three residual modules, and different data features were extracted using different-size convolution kernels. In the residual block, all three residual structures contained a convolutional layer, a maximum pooling layer, and an attention mechanism module. The processing of the spectral data was repeated twice in this architectural order to complete a residual block. To extract different data features using convolution kernels of different sizes, the parameters set were as follows. The input channels of the convolutional layer for all three residual modules were 64, and the convolutional kernel sizes were 3, 5, and 10, respectively, with a step size of 1. Multi-scale network structure can improve the ability of feature expression (Wu et al., 2023). For the maximum pooling layer, the kernel sizes were 2. The attention mechanism module used one-dimensional convolution to efficiently realize the local cross-channel interaction and extract the dependence between channels. After extracting the depth features through a series of convolution and pooling operations, the attention mechanism technology was used to adaptively weight the features of different information segments for information screening. At the same time, the screened features were re-labeled, and the weights of each channel were obtained by the Sigmoid activation function. The attention mechanism combined with the convolutional neural network can be used to capture the correlation between multiple variables and prediction results. It can enhance the effective information of hidden features extracted by each branch, suppress the invalid information, and assign the weights reasonably, which made it easier for the model to capture the interdependent relationship between spectral features. Therefore, adding the attention mechanism module can effectively improve the model efficiency and calculation effect. In this study, three kinds of residual blocks were constructed. When the data passed through three parallel residual blocks, identity mapping was used to avoid the disappearance of the network gradient. This structure can effectively reduce the gradient loss in the process of model learning, avoid the occurrence of degradation, and enhance the network learning ability.

Finally, the data was input to the Flatten layer, and the 1D multiple vectors were transformed into 1D single vectors through the Flatten layer and then inputted to the fully connected layer. To avoid overfitting the model, the Dropout layer was added to deactivate some neurons randomly. A softmax classifier was used to output the sample categories.

### 2.5 Software environment

The computer parameters for training and validation as well as the model environment resource configuration are listed in **Table 1**.

**Table 1:** Computer parameters and the model environment resource configuration.

Configuration	Parameter
Operating system	Windows 10
Development environment	Pycharm2019.3.3, MATLAB 2019b
Language	Python 3.7
Framework	keras 2.0.0, scikit-learn 0.21.3, tensorflow 2.2.0
GPU	NVIDIA GeForce RTX 3060
Accelerated environment	CUDA 11.1, CUDNN 11.1
CPU	AMD Ryzen 7 5800 8-Core Processor

### 2.6 Assessment standard of models

The purpose of model evaluation is to determine the effect of modeling methods. Different modeling methods will get different prediction results when modeling on the same data set. Compare and analyze the predicted situation and the real situation, and use the following methods to evaluate different algorithms quantitatively. Take the two-class confusion matrix, as shown in **Table 2**.

**Table 2:** An illustration of confusion matrix.

Real situation	Predicted situation	
	Positive example	Counter examples
Positive example	TP	FN
Counter examples	FP	TN

The short names in the table have the following meanings.TP: true positive, positive samples are classified as positive samples; FP: false positive, negative samples are classified as positive samples; TN: true negative, negative samples are classified as negative samples; FN: false negative, positive samples are classified as negative samples.

Accuracy refers to the percentage of discriminated correct results in the total sample;


(2) $$Accuracy=\frac{\left(TP+TN\right)}{\left(TP+FP+TN+FN\right)}$$

Precision refers to the probability of actually positive samples among all the discriminated positive samples;


(3) $$Precision=\frac{TP}{TP+FP}$$

Sensitivity refers to the probability of being discriminated as a positive sample among the actually positive samples;


(4) $$Sensitivity=\frac{TP}{TP+FN}$$

The F1-score(Chinchor, 1992) is to combine the performance of precision and sensitivity;


(4) $$F1-score=\frac{2\times Precision\times Sensitivity}{Precision+Sensitivity}$$

## 3 Results

### 3.1 Spectral characteristics

#### 3.1.1 Spectral features in the VNIR band range

**Figure 3:**
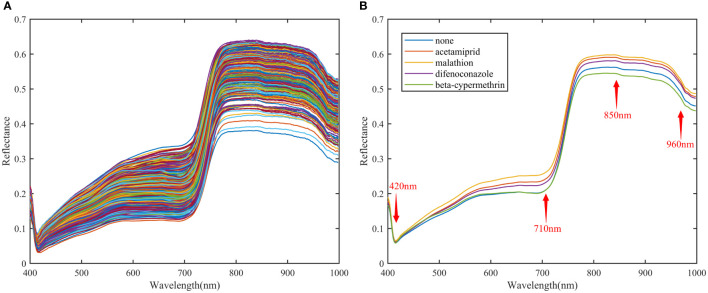
Raw spectrum and average reflection spectrum of VNIR. **(A)** Raw spectra; **(B)** Average reflectance spectra.

**Figure 3** includes the raw and average diffuse reflectance spectra of different types of pesticide residues on the surface of Hami melon in the band range of 400-1000 nm. **Figure 3A** shows the raw spectrum, and **Figure 3B** shows the average spectrum. As seen in **Figure 3**, the spectral reflectance of the different pesticide residues is different, but the trends are similar. Four main absorption peaks (420 nm, 710 nm, 850 nm, 960 nm) can be seen in **Figure 3**. Absorption peaks near 420 and 710 nm may be associated with carotenoid and chlorophyll absorption bands (Shao et al., 2020). Smaller absorption peaks are present near 850 nm, which may be three-order frequency doubling absorption characteristics of the C-H group (Sun et al., 2017; Hu et al., 2019). Absorption peak near 960 nm is associated with the moisture content of the Hami melon epidermis and may be second-order frequency doubling absorption characteristics of the O-H group (Gao et al., 2022).

As can be seen in **Figure 3B**, malathion has the highest average spectral reflectance in the entire band range. Taking 780 nm as the dividing point, the spectral reflectance of no pesticide residues and beta-cypermethrin are relatively close and have the lowest reflectance before 780 nm. After 780 nm, the average spectral curves of no pesticide residues and the four pesticide species are distinctly different. The average spectral reflectance of acetamiprid and diflubenzuron is slightly close to each other, near 970 nm. In addition, the differences between the mean spectra of beta-cypermethrin and the other spectra are most pronounced. The differences in the mean spectra provide a basis for identifying different pesticide residues on the surface of Hami melon.

#### 3.1.2 Spectral features in the SWIR band range

**Figure 4:**
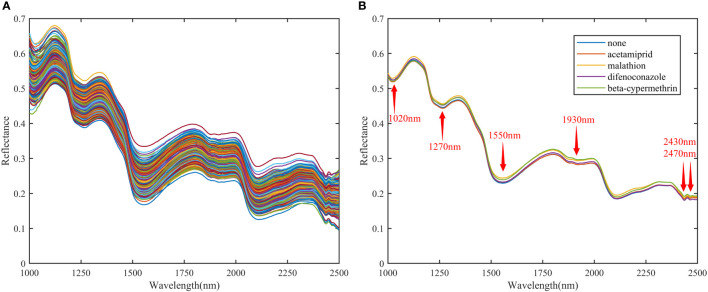
Raw spectrum and average reflection spectrum of SWIR. **(A)** Raw spectra; **(B)** Average reflectance spectra.

The raw and average diffuse reflectance spectra of different pesticide residues on the surface of Hami melon in the band range of 1000-2500 nm are shown in **Figure 4**. **Figure 4A** shows the raw diffuse reflectance spectrum, and **Figure 4B** shows the average diffuse reflectance spectrum. As with the results in the SWIR band range, the spectral reflectance of the different pesticide residues differ, but the trends in the spectral profiles are similar.

Specifically, in the 1493-2038 nm range, there were relatively significant spectral differences between samples with different types of pesticide residues and those without pesticide residues. The lowest reflectance was found in the 1490-1660 nm range for Hami melon samples without pesticide residues. The spectral reflectance of Hami melon samples sprayed with pesticides was higher than that of samples without pesticide residues, with the highest reflectance found for Hami melon samples sprayed with malathion. There were six absorption peaks in this spectral range (1020 nm, 1270 nm, 1550 nm, 1930 nm, 2430 nm, and 2470 nm). These absorption peaks may be associated with the periodic stretching vibrations of the C-H, O-H, and N-H bonds, which are the most fundamental bonds of organic compounds (Li et al., 2016a). 1020 nm is in the range of high absorbance and low reflectance, which corresponds to the second supersonic tone of the C-H stretching vibration (He et al., 2021). The band near 1270 nm may be associated with a second supersonic tone for C-H stretching in carbohydrates (Dong and Guo, 2015). 1550 nm is mainly associated with the chlorophyll absorption band of Hami melon and may also be related to the N-H first-order multiplication frequency (Sun et al., 2016a). The absorption peak for water is around 1930 nm, which is also the first overtone and combined mode of O-H group stretching (Siedliska et al., 2018). Absorption peaks at 2430 nm and around 2470 nm may be associated with the frequency synthesis of C-H and O-H groups (Sun et al., 2016b). However, these peaks and valleys cannot simply be used to distinguish between specific components, as the reflectance values at each band reflect complex compositional information. In particular, the spectra of organic pesticides were highly overlapping. In this study, the implied relationship between spectra and pesticide residues was discussed through data analysis.

### 3.2 Comparison of information fusion and single band detection effect

The 1D-CNN algorithm with attention mechanism was applied to analyze the spectral data in this stage in three band ranges. The results are shown in **Table 3** below. The spectral data in each band range were preprocessed with maximum-minimum normalization and smooth denoising before modeling. Comparing the identification results of the single-spectrum model and the fusion of spectral information, it can be found that the combined analysis using VNIR and SWIR was better. As can be seen from **Table 3**, among the detection results in a single band range, the accuracy of the single-spectrum model in VNIR achieved 84.50%, with the precision of 84.69%, recall of 84.50%, and F1-score of 0.8433; the accuracy of the single-spectrum model in SWIR achieved 89.00%, with the precision of 89.02%, recall of 89.00%, F1-score of 0.8890. The model built after data fusion had better performance on each criterion, with the accuracy of 94.00%, precision of 94.06%, recall of 94.00%, and F1-score of 0.9396. The accuracy of information fusion classification, which was poorly detected in a single band, was also improved after using information fusion modeling. Among them, malathion had the highest classification accuracy of 100.00%, which was an improvement over the two single band ranges.

**Table 3:** Classification performance of pesticide residues by single band range and information fusion.

Band(nm)	Class	Accuracy(%)	Precision(%)	Recall(%)	F1-score
400-1000	all	84.50	84.69	84.50	0.8433
	none	70.00	84.85	70.00	0.7671
	acetamiprid	92.50	78.72	92.50	0.8506
	malathion	95.00	92.68	95.00	0.9383
	difenoconazole	80.00	80.00	80.00	0.8000
	beta-cypermethrin	85.00	87.18	85.00	0.8608
1000-2500	all	89.00	89.02	89.00	0.8890
	none	75.00	83.33	75.00	0.7895
	acetamiprid	95.00	95.00	95.00	0.9500
	malathion	90.00	90.00	90.00	0.9000
	difenoconazole	95.00	84.44	95.00	0.8941
	beta-cypermethrin	90.00	92.31	90.00	0.9114
400-2500	all	94.00	94.06	94.00	0.9396
	none	90.00	90.00	90.00	0.9000
	acetamiprid	95.00	95.00	95.00	0.9500
	malathion	100.00	95.24	100.00	0.9756
	difenoconazole	97.50	92.86	97.50	0.9512
	beta-cypermethrin	87.50	97.22	87.50	0.9211

The two spectral ranges of VNIR (400-1000 nm) and SWIR (1000-2500 nm) showed great potential and good results for detecting different kinds of pesticide residues. The results of SWIR spectroscopy were slightly better than those of VNIR spectroscopy, with 4.50% higher accuracy, 4.33% higher precision, 4.50% higher recall, and 0.0457 higher F1-score in the SWIR band range than in the VNIR. Meanwhile, some scholars have also used the SWIR band to detect pesticide residues in fruits and vegetables and obtained good results (He et al., 2021).

Data layer fusion, as the most fundamental of fusion studies, is a direct fusion process of the data obtained from the sensors and a determination study based on the fused data. In this study, the spectral information obtained from the VNIR hyperspectral acquisition system (279 bands) and the spectral information obtained from the SWIR hyperspectral imaging system (233 bands) were united from the data fusion level. The data layer fusion completely preserved the spectral data information of the Hami melon surface in two different bands. Although the required data processing information was large, the convolution neural network can solve this problem well. And the F1-score of the model after information fusion was also improved, which showed that the model was of high quality. The proposed model can make better use of the different information from two spectral ranges to improve the classification performance.

### 3.3 Comparative analysis of the proposed 1D-CNN and traditional 1D-CNN

CNN, a feedforward neural network, is a crucial network structure in deep learning and has been successfully applied to qualitative and quantitative spectral data analysis. In this study, a custom 1D-CNN structure was proposed for classification to explore the potential information among spectral variables and to achieve the classification discrimination of different kinds of pesticide residues on the surface of Hami melon. The classification results of different pesticide residues using the traditional LeNet network and the proposed multi-branch 1D-CNN with attention mechanism are shown in **Table 4**.

**Table 4:** Classification performance of pesticide residues by LeNet and the proposed 1D-CNN model.

Model	Class	Accuracy(%)	Precision(%)	Recall(%)	F1-score
LeNet	all	89.50	90.10	89.50	0.8950
	none	87.50	83.33	87.50	0.8537
	acetamiprid	90.00	94.74	90.00	0.9231
	malathion	92.50	92.50	92.50	0.9250
	difenoconazole	97.50	82.98	97.50	0.8966
	beta-cypermethrin	80.00	96.97	80.00	0.8767
Proposed 1D-CNN	all	94.00	94.06	94.00	0.9396
	none	90.00	90.00	90.00	0.9000
	acetamiprid	95.00	95.00	95.00	0.9500
	malathion	100.00	95.24	100.00	0.9756
	difenoconazole	97.50	92.86	97.50	0.9512
	beta-cypermethrin	87.50	97.22	87.50	0.9211

**Figure 5:**
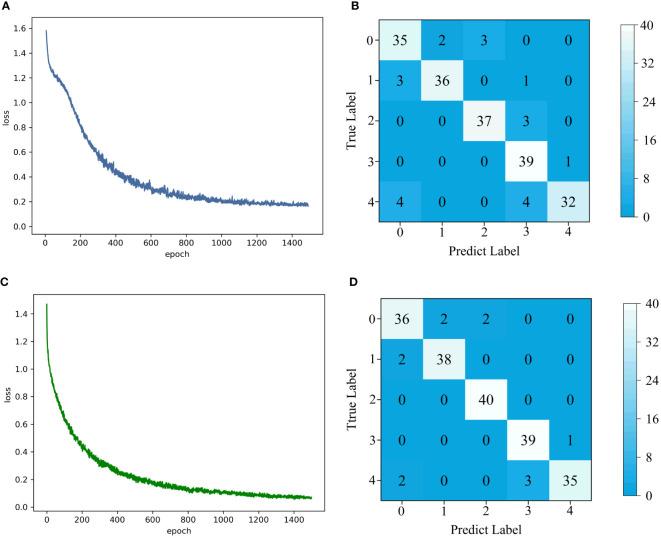
Loss function curve and confusion matrix of two CNN models. **(A)** Loss function curve of LeNet model; **(B)** Confusion matrix of LeNet model classification results; **(C)** Loss function curve of the proposed 1D-CNN model; **(D)** Confusion matrix of the proposed 1D-CNN model classification results.

Importing the spectral data into the model after information fusion, the accuracy of LeNet for pesticide residue classification was 89.50%, precision reached 90.10%, the recall was 89.50%, and F1-score was 0.8950. The proposed 1D-CNN model for pesticide residue classification showed 4.50% higher accuracy, 3.96% higher precision, 4.50% higher recall, and 0.0446 higher F1-score than LeNet. Among them, the accuracy of malathion and beta-cypermethrin improved the most by 7.50%. Difenoconazole had a 9.88% improvement in precision. The convergence curve and confusion matrix of LeNet and the proposed 1D-CNN model are shown in **Figure 5**. In **Figures 5A, C**, we can see that the loss curve of LeNet converges faster than that of the proposed 1D-CNN model, and the loss value is lower. To further explore the detailed classification performance of each level, **Figures 5B, D** show the confusion matrix of the classification results of two classification models, LeNet and the proposed 1D-CNN model, which were established by using 200 different spectra of the test set. In the confusion matrix, 0 in horizontal and vertical coordinates represents none, 1 represents acetamiprid, 2 represents malathion, 3 represents difenoconazole, and 4 represents beta-cypermethrin. As far as the overall recognition ability was concerned, the proposed 1D-CNN model misjudged only 12 samples, which was nine samples less than LeNet. In the LeNet model, beta-cypermethrin had the largest number of misjudged samples, with a misjudged rate of 20.00%, which were misjudged as none and difenoconazole, respectively, and the same number of misjudged types was four. Difenoconazole had the least number of misjudgments, only one misjudgment, and the misjudgment was beta-cypermethrin. In the proposed 1D-CNN model, the misjudgment degree of beta-cypermethrin was still the highest, with five samples misjudged (misjudgment rate: 12.50%). However, compared with LeNet, the misjudgment degree had decreased, which showed that the proposed 1D-CNN model could indeed improve discrimination accuracy. In addition, the discrimination of malathion was all correct, and there was no misjudgment.

### 3.4 Comparative analysis of the proposed 1D-CNN and traditional machine learning

**Figure 6:**
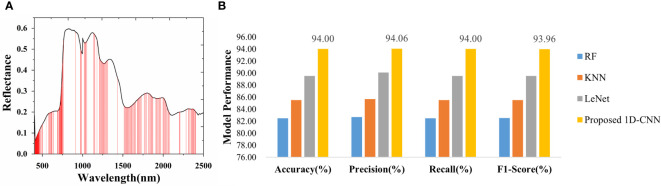
Characteristic bands and classification model results. **(A)** Distribution map of feature variables. **(B)** Test set results of four classification models.

Machine learning has been widely used in data mining, computer vision, natural language processing, and speech and handwriting recognition for qualitative or quantitative analysis. In this study, the spectral data after smooth denoising preprocessing were screened for feature bands, and a conventional GA was used for feature band screening. An appropriate feature selection method is beneficial to obtain more robust and accurate performance. **Figure 6A** shows the distribution of variables using the genetic algorithm. The red vertical solid line under the average curve reflects the extracted 179 effective bands. The number of spectral variables was reduced from 512 to 179 by GA feature bands, accounting for 34.96% of the entire 512 bands. The extracted feature bands were input into a traditional machine learning classification model for species identification.

The classification results of different pesticide residues using traditional machine learning and the proposed 1D-CNN model are shown in **Table 5**. Since the spectral data were nonlinear information, two commonly used nonlinear machine learning classification models, RF and KNN, were selected for detection, and the classification accuracies of both were higher than 80.00%. As shown in **Table 5**, the accuracy of RF was 82.50%, the precision was 82.70%, the recall was 82.50%, and the F1-score was 0.8251. Among the single class classification accuracy, the highest classification accuracy of malathion was 87.50%, and the lowest classification accuracy of difenoconazole was 77.50%. The accuracy of KNN was 85.50%, the precision was 85.67%, the recall was 85.50%, and the F1-score was 0.8553. Among the single class classification accuracy, the lowest classification accuracy of 77.50% was obtained for difenoconazole. The F1-score of KNN was 0.0302 higher than that of RF, indicating that the classification ability of KNN for different kinds of pesticide residues was better than that of RF.

**Table 5:** Classification performance of traditional machine learning and proposed 1D-CNN model for pesticide residues.

Model	Class	Accuracy(%)	Precision(%)	Recall(%)	F1-score
RF	all	82.50	82.70	82.50	0.8251
	none	82.50	84.62	82.50	0.8354
	acetamiprid	85.00	82.93	85.00	0.8395
	malathion	87.50	79.55	87.50	0.8333
	difenoconazole	77.50	77.50	77.50	0.7750
	beta-cypermethrin	80.00	88.89	80.00	0.8421
KNN	all	85.50	85.67	85.50	0.8553
	none	87.50	87.50	87.50	0.8750
	acetamiprid	87.50	81.40	87.50	0.8434
	malathion	87.50	85.37	87.50	0.8642
	difenoconazole	77.50	79.49	77.50	0.7848
	beta-cypermethrin	87.50	94.59	87.50	0.9091
Proposed 1D-CNN	all	94.00	94.06	94.00	0.9396
	none	90.00	90.00	90.00	0.9000
	acetamiprid	95.00	95.00	95.00	0.9500
	malathion	100.00	95.24	100.00	0.9756
	difenoconazole	97.50	92.86	97.50	0.9512
	beta-cypermethrin	87.50	97.22	87.50	0.9211

Deep learning has the advantages of strong learning ability and generalization ability. In this study, 1D-CNN with the multi-branch structure was used to extract spectral depth features of different levels and scales, which improved the ability of spectral data feature expression after information fusion. In addition, the model can extract spectral features by superimposing the convolution layer and pool layer in CNN and did not need preprocessing, such as feature band screening. The test set classification results of two traditional machine learning classification models and two deep learning models are shown in **Figure 6B**.

Meanwhile, it can be seen from **Table 5** that the F1-score of the proposed 1D-CNN model is 0.0843 higher than that of KNN, and the F1-score represents the harmonic mean of the precision and recall, indicating that the 1D-CNN model is more stable. Similar conclusions were obtained by related researchers in detecting grape hardness and PH value using VNIR, and deep learning had better prediction results compared to traditional machine learning (Xu et al., 2022a). In the field of pesticide residues, some scholars have used Raman spectroscopy to classify pesticide residue types and obtained better results using convolutional neural networks and transfer learning (Hu et al., 2022).

### 3.5 Model verification of the proposed 1D-CNN

In order to verify the accuracy of the proposed 1D-CNN model, we re-collected hyperspectral images of another batch of experimental samples. And 160 spectral data were extracted for experimental verification. The validation results of the model are shown in **Table 6**, in which the results of accuracy, precision, recall and F1-score are 92.00%, 91.27%, 92.00% and 0.9157, respectively. After calculation, the time complexity of the proposed model in this study was 37.15 M measured by FLOPs. The spatial complexity was measured by parameters of 0.39 M.

**Table 6:** Model verification results of the proposed 1D-CNN model.

Model	Class	Accuracy(%)	Precision(%)	Recall(%)	F1-score
Proposed 1D-CNN	all	92.00	91.27	92.00	0.9157
	none	90.00	85.71	90.00	0.8780
	acetamiprid	92.50	94.87	92.50	0.9367
	malathion	95.00	97.44	95.00	0.9620
	difenoconazole	95.00	88.37	95.00	0.9157
	beta-cypermethrin	87.50	89.74	87.50	0.8861

## 4 Discussion

HSI is a fast and non-destructive method to detect pesticide residues. In this study, VNIR, SWIR, information fusion and different classification models were used to identify the types of pesticide residues on the surface of Hami melon.

When VNIR and SWIR were used alone to detect pesticide residues on the surface of Hami melon, the accuracy of detection in the SWIR band was higher than that in the VNIR range of spectra, which may be due to the more pronounced peaks and valleys of the spectral profile in this band or to the greater reflectivity of this band for organic matter. Meanwhile, some scholars have also used the SWIR band to detect pesticide residues in fruits and vegetables and obtained good results (He et al., 2021). Compared with single-band detection, information fusion had a better detection effect. The information fusion data incorporated the differences and complementary information in the two spectra, resulting in a better differentiation than single spectral range detection. In addition, the peaks and valleys reflected by chemical groups in the two bands were different, reflecting different material information, which may be the reason why the classification effect was better after information fusion.

The extracted spectral data was preprocessed and then input into the classification models. Compared with the classification ability of LeNet, the classification ability of the proposed 1D-CNN model has been improved to some extent. The classification ability of the proposed 1D-CNN model has been improved to some extent. This may be because LeNet belongs to a single-branch neural network. Although it has some advantages for feature extraction ability, it cannot fully explore the internal connection of spectral data of different kinds of pesticide residues. And the single-branch CNN network may still have problems, such as loss of spectral feature information and disorder of structural information between data in the presence of a large amount of spectral information data. However, the 1D-CNN model proposed in this study was based on a three-branch parallel structure, and this multi-branch network structure can improve the performance and robustness of the model. Some researchers obtained similar results, and Li et al. (2022) also used a multi-branch architecture for fruit pedicel/calyx and defect identification of dried Hami jujube and obtained good detection results.

And three residual blocks of different scales were added to the multi-branch structure of this model. The number of independent paths was increased by using the residual structure to increase the dimensionality of the network. The multi-scale convolutional kernel was used to extract features to improve the nonlinear representation of the model and enhance the model’s performance. Besides, the increase in the number of layers in the network is usually associated with overfitting, gradient disappearance, and gradient explosion and consumes many resources. The residual structure used can solve the performance degradation problem caused by increasing the number of network layers. Zhang et al. (2019) also used a hierarchical fusion of residual networks to classify hyperspectral images and further improved the classification accuracy of hyperspectral images by adding a residual architecture.

In addition, the attention mechanism was introduced in the residual block to highlight important information in the spectral data rather than focusing on the full information of the spectral data. The attention mechanism was calculated directly based on the input data, which focused on the correlation between elements within the input spectral data and achieved the focus on the spectral data by highlighting the weights of important correlations. Peng et al. (2022) used the MobileNet V2 model for grape pest identification and added the Coordinate Attention (CA) mechanism to enhance the information characterization ability of the model, and the accuracy of identification on the grape pest dataset was 89.16%. Therefore, including the attention mechanism module can effectively improve the model’s efficiency and computational effectiveness.

Compared with the classification ability of traditional machine learning, the classification accuracy of CNN was more accurate. The reason for this may be that the spectral information of the two bands was fused in the data layer, the hyperspectral data was large and redundant, and the characteristic bands needed to be screened, so the preprocessing was complicated. Moreover, traditional machine learning is suitable for small sample learning and training, and the generalization ability is not high, so it is difficult to achieve the demand of classification accuracy using traditional machine learning classification methods.

In this study, the detection of different pesticide residues on the surface of Hami melon was realized by combining band information fusion with the proposed multi-branch 1D-CNN model with the attention mechanism. Subsequent research can try other deep learning models for classification, to obtain better results. This study provided convenience for the non-destructive detection of pesticide residues on the surface of large thick-skinned melons, thus promoting the development of food safety detection.

## 5 Conclusion

In this study, two hyperspectral imaging technologies with different spectral ranges and information fusion methods were used to successfully identify different types of pesticide residues on the surface of Hami melon. The classification performance of traditional machine learning and 1D-CNN for different kinds of pesticide residues was compared. Considering that the two spectral regions have complementary information, the spectral information of the two bands was fused, and a qualitative discrimination model of pesticide residues on the surface of Hami melon was developed. The results showed that the detection results of SWIR spectra were better than those of VNIR spectra. However, the fused information was better than the single band range detecting different pesticide residues. In this study, a multi-branch 1D-CNN model with the attention mechanism was proposed, which used a multi-branch structure to improve the performance and robustness of the model. Three different architectures of residual blocks were added to the multi-branch structure to address the performance degradation caused by increasing the number of layers in the network. The attention mechanism was introduced in the residual blocks to highlight important information in the spectral data. In addition, on the spectral data of information fusion, the proposed 1D-CNN model was tested and compared with the traditional machine learning classification models KNN and RF that applied the GA feature band screening method. The proposed 1D-CNN gave the best classification results with the accuracy of 94.00%, precision of 94.06%, recall of 94.00%, and F1-score of 0.9396. Overall, this study showed that HSI could be used as a non-destructive and efficient method to detect pesticide residues on the surface of Hami melon. The use of spectral information fusion and the 1D-CNN model can effectively improve the classification accuracy for different kinds of pesticide residues. In addition, this method can provide a reference for detecting pesticide residues in other fruits and vegetables. Furthermore, the types of pesticide residues on the surface of Hami melon differed from Hami melon varieties. Subsequent attempts will make to introduce other varieties of Hami melon and multiple pesticide species and increase the pesticide gradient settings to further validate the effectiveness of the method. In addition, an attempt will be made to further exploit the image information in the hyperspectral images to improve the performance of the prediction model through the fusion of spectral and image information. So as to realize non-destructive detection of the quality and safety of Hami melon. The combination of micro-hyperspectral and other techniques will also be explored to deeply explore the physicochemical properties of the samples and combine the fluorescence information of pesticides to more completely represent the effects of pesticide residues on Hami melon.

## Data availability statement

The original contributions presented in this study are included in the article/supplementary material. Further inquiries can be directed to the corresponding author.

## Author contributions

YH and YZ used the hyperspectral imaging system to obtain hyperspectral images. YH, HW and BM designed the experiment. YH wrote the manuscript. YH and YZ completed the development and implementation of methods. GY and YL made guidance for the writing of the manuscript. BM provided funding for this work. All authors contributed to the article and approved the submitted version
